# Professional Development and Scholarly Activity Weeks to Enhance the Master Adaptive Learner and Foster Future Educators

**DOI:** 10.1007/s40670-024-02027-7

**Published:** 2024-03-25

**Authors:** Britani Javed, Aaron Allgood, Breanne Jaqua

**Affiliations:** Clinical Education Department, A.T. Still University School of Osteopathic Medicine in Arizona, Mesa, AZ USA

**Keywords:** Master adaptive learner, Learning theory, Osteopathic medical education, Metacognition, Peer teaching

## Abstract

One osteopathic medical school designated 4 weeks per year in the first-year curriculum to Professional Development and Scholarly Activity. The aim of these weeks is to foster the development of Master Adaptive Learners. This metacognitive learning theory promotes adaptive expertise which supports students as educators in a variety of settings.

A.T. Still University, School of Osteopathic Medicine in Arizona reserved four weeks of the curriculum during the 2022−23 educational calendar to focus on Professional Development and Scholarly Activity (PDSA). During these weeks, first-year osteopathic medical students engaged in meaningful active learning sessions related to the principles of Master Adaptive Learning. The desired outcome from these dedicated weeks was the development of professional knowledge, skills, and attitudes that would transfer to students’ experiences inside and outside of the classroom. Outcomes were measured using pre- and post-surveys related to the key characteristics of the Master Adaptive Learner. The innovation described here adds to the growing body of literature related to the application of Master Adaptive Learning within the realm of medical education, and specifically how medical schools can support students in their development as Master Adaptive Learners and ultimately future educators.

Each PDSA Week occurs at the beginning of every 10-week educational block. The longitudinal content throughout the four PDSA Weeks focuses on developing the four personal characteristics of the Master Adaptive Learner: Curiosity, Growth Mindset, Motivation, and Resilience [1]. Sessions during the PDSA Weeks are led by faculty, staff, and senior administrators on a variety of topics including scholarship, professional identity formation, medical ethics, academic advising, interprofessional education, wellness, humanities, and diversity, equity, and inclusion. The topic-focused sessions, lectures, and workshops that existed elsewhere in the curriculum were coalesced into these distinct PDSA Weeks; as such, there was no loss of overall time for the basic sciences curriculum. To evaluate the effectiveness of the curriculum, students completed a voluntary and anonymous pre- and post-survey at the beginning and end of each PDSA Week, respectively. The A.T. Still University-Arizona Institutional Review Board granted the study exempt status (IRB no. 2022 − 103).

The pre- and post-survey format was used, and the survey statements were derived from previously validated sources. Survey statements were selected using a panel of educators that provided expert opinions at our institution. A list of the survey statements is available in Table 1. The responses used a 5-point Likert scale demonstrating students’ level of agreement with the statements. Full data analysis is pending. The first PDSA Week of the academic year was not included in the data collection, as the grant associated with this innovation was not issued until after the start of the academic year. Preliminary results from PDSA Weeks 2, 3, and 4, indicate high self-reported levels of curiosity, growth mindset, motivation, and resilience in both pre- and post-survey responses.

**Table 1:** List of the anonymous pre-and post-survey statements

Curiosity I enjoy exploring new ideas.^a^ I am always looking for experiences that challenge how I think about the world and myself.^b^ I view challenging situations as an opportunity to grow and learn.^b^
Resilience I look for creative ways to alter difficult situations.^c^ I believe I can control my reaction to events that happen both to me and around me.^d^ It is hard for me to snap back when something bad happens.^e^
Motivation I’m concerned about taking on a task at work if my performance would reveal that I have low ability.^f^ I would avoid taking a new task if there was a chance that I would appearincompetent to others.^f^ I try to figure out what it takes to prove my ability to others at work.^f^
Growth Mindset I recognize my need to constantly acquire new professional knowledge.^g^ I enjoy challenging and difficult tasks at work where I’ll learn new skills.^f^

^a^[Adapted from] Naylor FD. A state-trait curiosity inventory. *Aust Psychol.* 1981;16(2):172-183. doi: 10.1080/00050068108255893

^b^Kashdan TB, Gallagher MW, Silvia PJ, et al. The curiosity and exploration inventory-II: development, factor structure, and psychometrics. *J Res Pers*. 2009;43(6):987-998. doi: 10.1016/j.jrp.2009.04.011. PMID: 20,160,913

^c^Sinclair VG, Wallston KA. The development and psychometric evaluation of the brief resilient coping scale. *Assessment*. 2004;11(1):94-101. doi:10.1177/1073191103258144

^d^[Adapted from] Sinclair VG, Wallston KA. The development and psychometric evaluation of the brief resilient coping scale. *Assessment*. 2004;11(1):94-101. doi:10.1177/1073191103258144

^e^Smith BW, Dalen J, Wiggins K, Tooley E, Christopher P, Bernard J. The brief resilience scale: assessing the ability to bounce back. *Int J Behav Med.* 2008;15(3):194-200. doi: 10.1080/10705500802222972

^f^Vandewalle D. Development and validation of a work domain goal orientation instrument. *Educ Psychol Meas*. 1997;57(6):995–1015. doi: 10.1177/0013164497057006009

^g^Wetzel AP, Mazmanian PE, Hojat M, et al. Measuring medical students’ orientation toward lifelong learning: a psychometric evaluation. *Acad Med*. 2010;85(10):S41-S44. doi:10.1097/ACM.0b013e3181ed1ae9

Given this initial observation, we wonder if perhaps there is selection bias, as students who chose to come to our institution for their undergraduate medical training were informed about the nature of the self-driven, case-based curriculum prior to matriculation. As such, students who are interested in becoming Master Adaptive Learners may have self-selected into the medical school compared to those desiring a more traditional, lecture-based curriculum. It is possible that students seeking a problem-based learning style have high intrinsic curiosity, growth mindset, motivation, and resilience.

We believe that by fostering the knowledge, skills, and attitudes of the Master Adaptive Learner, students will become better educators. At its core, Master Adaptive Learning theory promotes the development of adaptive expertise [1], which will support students as they progress to various types of educational roles including teaching patients, peers, and colleagues. Taking into account the continuum of medical education, residents-as-teachers is an established paradigm wherein senior trainees engage in peer education of junior trainees [2]. In healthcare settings where teaching occurs in a fast-paced, complex environment, this learning theory is ideal because adaptability is one of the key tenets [1]. Furthermore, Master Adaptive Learners must develop robust metacognitive skills [1], which can be translated to clinically adjacent tasks.

Our vision is to extend the PDSA training to all four years of undergraduate medical education at our institution. We are in the process of analyzing data, and will reflect on this year’s work to determine areas of future study and how to refine the curriculum. Our goal is to have a finalized educational calendar of curricula that is proven to be effective in educating students on how to become Master Adaptive Learners.

## References

[1] Cutrer W, Pusic M, Gruppen LD, Hammoud MM, Santen SA. The Master Adaptive Learner. Elsevier; 2020.

[2] Baser-Decker T, Ellis S, Bartlett H (2000). Applying adult learning theory to a residents-as-teachers workshop series. Acad Med.

